# Issues and Perspectives in Meditation Research: In Search for a Definition

**DOI:** 10.3389/fpsyg.2012.00613

**Published:** 2013-01-10

**Authors:** Bhuvanesh Awasthi

**Affiliations:** ^1^Department of Cognitive Science, Macquarie UniversitySydney, NSW, Australia

**Keywords:** meditation, definition, neural correlates, consciousness, contemplative practice

## Abstract

Despite the growing interest in the neurobiological correlates of meditation, most research has omitted to take into account the underlying philosophical aspects of meditation and its wider implications. This, in turn, is reflected in issues surrounding definition, study design, and outcomes. Here, I highlight the often ignored but important aspect of definition in the existing scholarship on neuroscience and meditation practice. For a satisfactory account of a neuroscience of meditation, we must aim to retrieve an operational definition that is inclusive of a traditional ontological description as well as the modern neurocognitive account of the phenomena. Moving beyond examining the effects of meditation practice, to take a potential step forward in the direction to establish *how* meditation works, it becomes crucial to appraise the philosophical positions that underlie the phenomenology of meditation in the originating traditions. This endeavor may challenge our intuitions and concepts in either directions, but issues pertaining to definition, design, and validity of response measures are extremely important for the evolution of the field and will provide a much-needed context and framework for meditation based interventions.

## Introduction

In recent years, research on neural correlates of meditation and its efficacy in clinical settings has seen a growing trend. Meditation training has been associated with a wide range of positive clinical and behavioral health outcomes and several researchers have identified key areas of the brain and other electrophysiological correlates of both novice and long-term meditators. The study of expert meditators is believed to offer a promising research strategy for studying high-order cognitive processes.

Despite a laudable attempt to ascertain neural markers of meditation practice and a suggested importance in informing us about the neural bases of consciousness (Braboszcz et al., 2010), a number of issues and shortcomings remain in this area of research. This may be due to a variety of reasons, ranging from simply omissions, to a lack of suitable resources to address these issues, or relative unfamiliarity with the concept of meditation as discussed in the traditional texts.

A wide variety of techniques termed as “meditation” and a lack of agreement on how to best design meditation studies reflect a poor understanding of the ontological bases of meditation. In this context, Rao (2011) has argued that part of the problem is that meditation research is being carried out with little or no understanding of the theoretical and cultural nuances of meditation.

On one hand, meditation research presents an encouraging trend for a relatively new aspect of enquiry into how the mind works. On the other hand, accurate characterization of meditation theory and practice, in particular the fundamental issues surrounding definition, has escaped the attention of most meditation research published in contemporary research journals. In the relative absence of a mature and well-developed theoretical methodological foundation, the study of meditation owes a great debt to the contemplative traditions. This article aims to bring to notice the often ignored but important aspect of definition in the existing literature on neuroscience and meditation practice. It is critical not to generalize regarding the definition, neural correlates and effects of “meditation.” This is a call for an increased emphasis to carefully distinguish between the different techniques and phenomenologically defined features of each procedure being studied.

The purpose is not to discredit or call into question the neuroscientific findings, but to point to the import of those findings, together with the philosophical framework of meditation practice, in order to stimulate scientific and philosophical debate. Additionally, it should be noted that the focus here is not on providing a definition from either the traditional or the neuroscientific perspective. The aim, instead, is to point to the missing ontological grounding from which arise the issues surrounding definition and design that plague this area of research. To illustrate this point, I will briefly explore some reports that discuss the neural markers of meditation practice and highlight some mixed findings from these reports. I further argue that the issue of definition needs to be adequately addressed, failing which, several conflicts in the philosophical context of meditation practice vis-à-vis the neuroscientific search for the neural basis of meditation are likely to pile up.

## Definition Issues

A key concern in neuroscientific investigations of meditation processes is the lack of an operational definition. Can meditation be defined in scientific terms and if yes, why has it been neglected for so long? Additionally, if such an attempt is to be made, should the traditional definitions be ignored or reconciled with the modern neuroscientific understanding of mind and mental states? When the neuroscientific studies report their findings, are the studies reporting processes that are the epiphenomena of the meditation state or the actual neurophysical instantiation of the meditation practice? Are there several levels or states of meditation or is there just one state that is measured by electrophysiology, behavioral, and neuroimaging methods? Further, if there are several levels and/or states of meditation, do they all have similar or different instantiations or associated mechanisms in the brain? These are some of the questions that meditation research, presently, seems to have neglected.

Many reviewers and researchers practically assume that all processes labeled meditation are similar – an assumption which is flawed. At times, no distinctions are made between the various techniques and the different stages in the progress of meditation in the studies conducted. Without evaluating the differential effects of such techniques, a comparison of results from such comparative studies may not offer much in terms of characterizing the outcomes and alleged benefits (Rao and Paranjpe, 2008). At the same time, it is assumed that researchers and readers are well-versed with the state of meditation. Unlike the alert and wakeful state, meditation requires specific training. It is, therefore, difficult to separate the meditation state from the training that produces it (Tang et al., 2012).

In the modern context, meditation has been defined in a variety of ways, including attentional training to mindfulness (Kabat-Zinn, 2003) and relaxation-based methods (Benson et al., 1974) to automatic self-transcending (Travis and Shear, 2010). Some research suggests that different forms and practices can be seen as variations of concrete operationalizations of meditation (Bærentsen et al., 2010). A section of research studies continue to label a wide variety of mental training techniques as meditation, including, for example, imagery of the Buddhist deity (Kozhevnikov et al., 2009), *Hatha Yoga*, *Omkar meditation* (Harinath et al., 2004), *mantra meditation*, *Yoga*, *Tai Chi*, *Qi Gong* (Ospina et al., 2008), *brain-wave vibration meditation* (Jang et al., 2011), and *Kirtan Kriya* (KK) *meditation* (sound repetition with finger-thumb touching; Moss et al., 2012). Others, like Baijal and Srinivasan (2010), studied a concentrative form called *Sahaj Samadhi meditation*, while Vago and Nakamura (2011) studied the *Mindfulness-based Meditation Training* (MMT) program involving a combination of concentrative and open monitoring (OM) types of meditation, breath and body awareness, light stretching, and relaxation exercises.

Ospina et al. (2007) meta-analyzed a large sample of studies within five broad categories of meditation practices (*mantra meditation*, *mindfulness meditation*, *Yoga*, *Tai Chi*, and *Qi Gong*). Reporting poor methodological quality in the studies examined, they suggested that positive therapeutic effects and well-being may not be specific to the meditation practices and can be achieved by somatic relaxation training and prayer. In contrast, Hofmann et al. (2010) argued for specific support of mindfulness-based therapy for treating anxiety and depression. In another recent study, Luders et al. (2012) showed evidence for thicker corpora callosa in meditators compared to controls. The meditators in this study (age range: 24–64 years) had been practicing from 5 to 46 years and were recruited from Chenrezig, Kriya, Shamatha, Vajrayana, Vipassana, and Zazen traditions. While Luders et al. (2012) argued that the inclusion of various meditation styles allows for an investigation of the neural correlates of common and overlapping elements of meditation, detailed phenomenological as well as neuro-behavioral accounts of the various styles remains to be seen in the literature.

## Meditation as Relaxation

In the early days of research on meditation, relaxation was the key conceptual factor. Relaxation response was proposed as a physiological state opposite to that of stress, aiming to modify mental activity, and reduce arousal as a kind of therapeutic intervention. In contrast to attention training or (Benson’s) relaxation response, some traditional texts describe meditation as the elimination of thought activity (Rukmani, 2001), i.e., mental silence that is orientated toward a specific/altered state of consciousness. Without reducing alertness, the practice aims to eliminate mental activity altogether as part of an overarching strategy to facilitate the development of consciousness. The “relaxation” conceptualization of meditation thus completely ignores the concept of mental silence or “trans-thought awareness” (Krishnamurti, 1975, p. 216; Osho, 1996).

## Meditation as Attention

While early studies ascribed the practices of relaxation and focusing attention to meditation, a few recent attempts have been made to delineate the specific psychological processes implicated in these two practices and to derive neurofunctional predictions. Within the attentional paradigm, Dunn et al. (1999) and Lutz et al. (2008) have proposed two broad categories: focused attention (FA) and OM type of meditation. These, according to the authors, are based on traditional meditation texts and modern neuroscientific conceptions. However, it is likely that different traditional texts may word the description differently depending on context, language, and available translations. Further, Travis and Shear (2010) have argued for self-transcending, whereas Rao (2011) suggests passive diffused attention or “inattention” as the defining feature of meditation. Recently, Mikulas (2011) has reported that mindfulness and concentration practices are often confused in the literature. Therefore, a theoretical framework is needed to produce an operational definition for meditative practices that can be adopted in the scientific study of effects of meditation training on the brain. While the FA versus OM distinction represents a considerable advance over assumptions of equivalence, and may be a productive distinction in some contexts, it represents only an initial stage of stimulus description and analysis. For instance, Bærentsen et al. (2010) quote a classic text to describe meditation:

“meditation is defined as *the control of fluctuations of the mind* that aim to *still the fluctuations (patterning) of the mind*. When the fluctuations of the mind are controlled, the yogi achieves concentration, i.e., meditation (samadhi or Nirvana).” (p. 58, italics in original)

The authors (p. 58, italics in original) then continue to describe meditation “in contrast to a ‘normal,’ uncontrolled state of mind in which, according to Patanjali, *the self (the mind, awareness) identifies itself with the fluctuating patterns of consciousness*.” Here, it should be noted that concentration and meditation are used interchangeably and several different concepts and terminologies like *samadhi*, *nirvana*, and fluctuations of consciousness are being introduced. This ambiguity in defining the task at hand probably also stems from a reliance on unverified translations of the original classical texts with no comparable concept available in neuroscience. Some of these issues, as Rao (2011) notes, can be attributed to borrowed concepts and tools lacking in relevant conceptual clarity and methodological sophistication.

Patanjala-Yoga-Sutra (PYS), a seminal text, describes concentrative focus as “*dharana*” and effortless awareness “*dhyana*” as distinct states. Other texts also differentiate between the focused attentional training (*dharana*), and the meditative state of *dhyana* (*GoraksaSatakam*: Kuvalayananda and Shukla, 2006; *Gheranda Samhita*: Digambarji and Gharote, 1997). In a scholarly commentary on the PYS, Karambelkar (2006) reported several stages of concentrative focus or *dharana*. *Dhyana* or meditation is described as a very precise state that goes beyond (transcends) the focused one-pointedness. It is also mentioned that the former *dharana* (concentration), the middle *dhyana* (meditation), and final *samadhi* (absorption) are consecutive stages and that the earlier ones transform and culminate into the next. Thus, it should be clear that the technique (imagery, chanting, or breath watching), states produced (concentration, attentive, OM, or mindfulness), and the mechanical dynamics (fluctuations of mind) underlying different types of experiences need to be distinguished. One should bear in mind that the list of techniques and states discussed here is not exhaustive. With an exclusive focus on assessing cognitive capacities of attention and awareness, investigation of meditation and its effects shall remain confined to particular domains.

## Meditation as Mindfulness

More recently, mindfulness has been proposed as a cognitive behavioral, rather than physiological, paradigm for meditation. Mindfulness aims to develop enhanced awareness of the moment-to-moment experience of perceptible mental processes. It involves “training practitioners to attend to a wide range of changing objects of attention while maintaining moment-to-moment awareness (mindfulness), rather than restricting one’s focus to a single object such as a mantra” (Kabat-Zinn, 2003, p. 145). Several mindfulness-based interventions, such as the Mindfulness-Based Stress Reduction (MBSR: an 8-week program with 3 h of weekly group sessions and daily homework), have since been developed for clinical and empirical research on the topic. Mindfulness and concentration are not necessarily contrasted as distinct practices (in some Buddhist cases concentration meditation requires mindfulness) and diverse perspectives on the meanings and origins of mindfulness have been offered by various researchers (Williams and Kabat-Zinn, 2011).

Grossman (2008, 2011), Van Dam et al. (2009), and Grossman and Van Dam (2011) have called into question whether self-report measures of mindfulness [Mindful Attention Awareness Scale (MAAS) and Mindful Attention Awareness Scale – Adolescent (MAAS–A)] actually assess mindfulness. There are many different scales that purport to measure mindfulness and the results obtained reflect more about the scales, rather than mindfulness itself (Van Dam et al., 2011). Assessing constructs in a culture different from where it originated is likely to have limitations. In the relative absence of external referents to verify the validity of constructs of mindfulness use in self-report scales, much of the research findings based on these indirect assessments remain limited in its validity. In the traditional context, mindfulness is derived from the Pali word “*sati*” that conveys the meaning-*to remember* (remember to maintain awareness) with four distinct phases described in the traditional literature. This is clearly distinct from the modern attempts at operationalizing a fixed trait-like definition of mindfulness that ignores the developmental and contextual aspects (Grossman and Van Dam, 2011). Individuals with no meditation experience respond to the word “mindfulness” questionnaire items differently from people with meditation experience, as seems likely from the results of a study (using the MAAS), where binge-drinking college students scored significantly higher scores compared to experienced meditators (Leigh et al., 2005).

It was further queried if the construct of mindfulness can be understood apart from mindfulness training, and whether there is empirical evidence to support the validity of mindfulness measures. More such issues regarding reliability and validity of self-report questionnaires have been raised recently (Bergomi et al., 2012; also see Brown et al., 2011). In assessing all the available self-report scales of mindfulness, Bergomi et al. (2012) report that these scales do not offer a comprehensive assessment of all aspects of mindfulness in samples from the general population. Similarly, Chiesa (2012) stated that modern attempts to operationalize mindfulness have consistently failed to provide an unequivocal definition of mindfulness that takes into account the complexity of the original traditional definitions of mindfulness. According to Grossman (2011), currently used self-report measures of mindfulness may instead reduce and distort the meaning of mindful awareness in psychological sciences that could negatively impact the possibility of further development of mindfulness-based interventions.

## Mixed Findings

A growing body of research has investigated the effects of meditation practice on the resting or “default” state, with a wide range of findings reported. Default-mode network (DMN) includes the medial prefrontal cortex (mPFC), anterior cingulate cortex (ACC), and posterior cingulate cortex (PCC), with all these regions active in the resting state. Some studies have reported enhanced functional connectivity and/or resting state activity in the frontal cortices associated with several different meditation practices (*Mindfulness meditation*: Berkovich-Ohana et al., 2012; *Zen meditation*: Faber et al., 2008; *Brain-wave vibration meditation*: Jang et al., 2011; *Mindfulness-Based Stress Reduction*: Kilpatrick et al., 2011). Cortical areas associated with the DMN are substantially similar to those associated with mind wandering (Gusnard et al., 2001; Kelley et al., 2002; Buckner et al., 2008) and thus have been implicated in meditation practice.

Jang et al. (2011) studied the effects of “*brain-wave vibration meditation*” (a kind of moving meditation to quieten the thinking mind and release negative emotions by performing natural rhythmic movements and focusing on bodily sensations) and report increased greater functional connectivity within the DMN in the mPFC area compared to controls. In contrast, in an fMRI study exploring functional connectivity in *Zen meditators*, Taylor et al. (2012) report weaker functional connectivity between DMN regions in meditators compared with controls. In another fMRI functional connectivity study by Kilpatrick et al. (2011), compared to controls, subjects with 8 weeks of *MBSR training* showed significant functional connectivity differences in auditory and medial visual networks. In a study with three meditation types – concentration, loving-kindness, choiceless awareness, Brewer et al. (2011), found that the main nodes of the DMN (medial prefrontal and posterior cingulate cortices) were relatively deactivated across the three meditation types studied. In a more recent fMRI study involving functional connectivity analyses, Froeliger et al. (2012) report that *mindfulness meditation* practitioners exhibit significantly greater resting state functional connectivity in DMN than control subjects. In contrast, in an EEG study by Berkovich-Ohana et al. (2012), mindfulness meditators exhibited lower DMN activity compared to controls. The conflicting results in these studies could be due to varied reasons like different techniques studied or subjects tested in different stages of meditation.

Thus, it is obvious that different kinds of meditation techniques show different functional connectivity patterns in the DMN. Given the variety of techniques clubbed together as meditation, these findings are difficult to interpret and remain puzzling in the context of a lack of consensus on the definition of meditation/a particular meditation. One of the reasons that researchers get different results is because they are studying different procedures. Different techniques of meditation employing a wide variety of methods like mental imagery, chanting, concentration training, OM, or breath counting etc., are expected to exhibit different patterns of functional connectivity.

## Study Design Issues

For an objective examination of the correlates of any stimulus or mental task or phenomena on behavioral or neurological changes, the characteristics of the stimulus in question have to be tightly controlled and characterized with utmost precision. The matter of the sensitivity, appropriateness, reliability, and validity of response measures is extremely important for the evolution of any research field, and especially for meditation research. Incorporating the ontological definition of meditation will provide a much-needed context and framework for the interventions borrowed from or based on meditation. The lack of an operationalized definition further leads to several study design issues.

A significant number of studies on meditation that aim to explore its neural concomitants have largely employed protocols that ignore comparisons with the non-meditator control participants (Wallace, 1970; Hebert and Lehmann, 1977; Aftanas and Golocheikine, 2001; Arambula et al., 2001). Some studies have compared meditators with non-meditators during rest (Travis et al., 2002; Davidson et al., 2003). Trait effects occur due to a long-term practice of meditation resulting in baseline differences between meditators and non-meditator controls (Cahn and Polich, 2006). Results from studies (Deepak et al., 1994; Khare and Nigam, 2000; Aftanas and Golocheikine, 2001; Travis et al., 2002) that have compared neural activity during meditation with the resting state of meditators may be confounded by the trait changes in meditators. The studies that compare oscillatory activity between meditators (during meditation) and non-meditators (during relaxation; Banquet, 1973; Lehmann et al., 2001; Travis et al., 2002; Davidson et al., 2003) have also ignored the tracking of state changes that occur with meditation practice. Travis (2011) explored if psychological well-being and mindfulness are related to the type of meditation technique practiced and argued that it was difficult to interpret findings between different groups due to large demographic and process differences.

Through a meta-analysis, Sedlmeier et al. (2012) provided a comprehensive overview of the effects of meditation on psychological variables in non-clinical groups of adult meditators. The authors identified several methodological problems in a large number of studies and reported a lack of sufficient theoretical background in most studies. They examined 21 separate categories of dependent measures in a large number of meditation research reports and reported that overall, meditation does not exert uniform effects on the categories of dependent measures examined. The authors compared meditation with relaxation response and cognitive training and reported that meditation is not just a relaxation technique. Instead, they found that meditation has a substantial impact on psychological variables, and these effects are stronger for emotional than for cognitive variables.

## Technique Versus State

Many researchers also seem to confuse the *technique* of meditation with the *state* of meditation. A large variety of research variously describes several techniques and calls them different meditations. The traditional texts describe a multitude of techniques, with anthologies discussing eligibility and suitability for enthusiasts and students, outlining the different types, tastes, and expertise levels. For instance, just one text, *Vigyan Bhairav Tantra* describes over a hundred different meditation methods that range from silent sitting, breath observation, and mental imagery to vigorous chaotic breathing, intense activity, and sexual excitation. If meditation is a mental state, precise characteristics of the state need to be identified. Holding an image of some deity, flowing compassion toward the whole of humanity, or concentration are mental activities, or mental states. The issue then is, how are these mental states different from any other mental states, such as doing arithmetic, or remembering past events? Is meditation a different (unique) kind of mental state? Or is it a stance – of non-judgment, watchfulness, and non-identification toward any and all thought activity? If there are techniques involved in attaining or leading up to the states of meditation, all the steps involved in the process need to be outlined. Different techniques employed in different meditative practices will have different neurophysiological correlates. The failure to delineate meditation techniques from the meditative state thus confounds research findings.

Despite the fact that previous research has proposed a distinction in trait versus state measurement of meditation effects, a sizable number of current studies treat mindfulness as a trait-like quality. Contending that trait versus state classification of mindfulness is not mutually exclusive, Chiesa (2012) argues that mindfulness can also be considered a state that is maintained when attention to experience is intentionally cultivated (also see Thompson and Waltz, 2007). In such a scenario, it becomes pertinent to examine how mindfulness is conceptualized in a particular study and the psychological variables used to measure the efficacy.

It is often overlooked that most meditation techniques in the originating disciplines were not promulgated as stress reduction methods alone. In many traditional contexts, a wide variety of preparatory measures and practices were considered essential before being introduced to meditation techniques. There are many techniques, involving body postures and activities, mental imagery to sounds, and chanting accompanied by fragrant and calm environs, that may facilitate certain brain changes.

The preparatory methods comprised several stages within disciplines like Yoga (consisting of many steps, including moral and psychological training, posture practice, breath modulation, diet, and behavioral recommendations, among others) as described in PYS (Karambelkar, 2006), Hathapradipika of Swatmarama (Digambaraji and Kokaje, 1998), GoraksaSatakam (Kuvalayananda and Shukla, 2006), Gheranda Samhita (Digambarji and Gharote, 1997), and Shiv Samhita (Maheshanandji et al., 1999). These preliminary practices were followed by relaxation techniques, mediated through the autonomic nervous system, which then prepared a subject for introduction to meditation techniques. While the techniques by themselves are quite varied and aim to bring about an attentive state of OM of physiological and mental events without judgment, it bears repeating that the techniques themselves are different from the state. In such a scenario, it may be pertinent to tease apart the relaxation changes brought about by techniques and the neuro-behavioral changes associated with the state of meditation itself, and studies should clearly identify them as such. The failure to distinguish between the state versus the technique of meditation contributes to the confusion in the field (Rao, 2011).

## One Size Fits All?

Meditation is commonly promoted as a way to reduce stress, bring about relaxation, and even manage mental health issues like depression. There are widespread courses conducted for classrooms, prisons, and hospitals with the underlying belief and assumption that it is good and safe for everyone and that most people are ready for it. While a majority of meditation teachers with varied techniques, prescribe meditation for a wide variety of psycho-spiritual ailments, it remains to be seen if the techniques on their own bring about expected results. While basic techniques like watching the breath may not be complicated for many, others that include vigorous activity, sound based, or mental imagery, might not be equally risk-free. A number of studies have documented negative side-effects induced by meditation and relaxation-based methods (*Transcendental meditation on blood pressure*: Canter and Ernst, 2004; *Relaxation-associated panic attacks*: Cohen et al., 1985; *Relaxation-induced anxiety*: Heide and Borkovec, 1983, 1984; *depersonalization syndrome*: Kennedy, 1976; *Relaxation-induced panic attacks*: Lazarus and Mayne, 1990; *Spiritual practices and DSM-IV*: Lukoff, 1998; *Adverse effects in long-term meditators*: Shapiro, 1992). Thus, in addition to finding an appropriate technique, safety, and efficacy also remain important starting points before meditation is publicized as a panacea for restoring mental health and well-being. It would be worthwhile to point out that in the modern setting, while meditation is being proposed (and taught in clinical settings) to bring about physical well-being, mental health, and to enhance cognitive and other skills; in the traditional context, however, physical and mental well-being are *pre-requisites* to begin meditation practice.

Recently, in a meta-analysis, Eberth and Sedlmeier (2012) reported large differences in the effect sizes reported for MBSR versus mindfulness meditation. The authors argued that the reported effects of MBSR over mindfulness might be due to additional factors such as psychoeducation, participants’ expectations, as well as methodological variations in the studies comparing MBSR and mindfulness. Other reasons include a focus on psychological health rather than higher mental states, and differences between the participant groups (people attending MBSR courses for stress reduction might differ from people visiting a meditation center to attain wisdom or higher mental states).

Another aspect of meditation research involves measuring the quantity or dosage of meditation. Various studies report that meditation-related benefits are associated with the amount of practice a person has undertaken (with the aim that repeated sustained practice leads to beneficial effects; *Concentration meditation*: Brefczynski-Lewis et al., 2007; *compassion meditation*: Pace et al., 2009; *any meditative tradition (unspecified)*: Baron Short et al., 2010; *FA*, and *OM*: Manna et al., 2010). A number of studies report the total number of years, while others detail the total hours (usually in thousands) that a meditator has engaged in meditation. Comparing meditators (from FA and OM styles) with controls, Chan and Woollacott (2007) report that the amount of time spent meditating each day is a stronger predictor of attention performance, compared to the greater number of hours of meditative practice over a lifetime. Hasenkamp and Barsalou (2012) went a step further and reported the total lifetime hours (number of years × days × minutes each day, including retreats) of FA meditation experience. This quantification, though useful, assumes that meditation is a single state wherein the duration of time spent practicing amounts to meditation experience. Such quantification ignores the concept of depth of meditation as well as the possible levels of depth involved in the experience.

In a study of sustained attention-based meditation, Brefczynski-Lewis et al. (2007) reported that compared to novices, expert meditators showed higher brain activation with 19,000 h of practice, while with 44,000 h, they showed lesser activation. Recently, there have been some reports on the efficacy of short-term meditation on brain function (Tang et al., 2007, 2010; Barnhofer et al., 2010; Moyer et al., 2011). A number of traditional meditation practices are known to have demanded dedicated effort and time for several years to obtain and maintain a steady, detached, witnessing state of mental functioning (Thera, 1973; Gunaratana, 2002). It still needs to be established how the expectations of immediate and quick results collate with the long-term commitment for dedicated, continued practice in the traditional context. This would enable some guidelines regarding an efficacious dosage for therapeutic purposes in clinical settings. As of now, no unique behaviors and neurophysiological patterns have been documented to be specific to meditation, making it difficult to gage the extent to which meditation practice changes behavioral or neural structure. Rao (2011) has argued that current meditation research focusing solely on (short) term practice and efficacy of meditation for health benefits may be mistaking the chaff for the wheat.

Forsaking the important issue of defining the terms properly in a neuroscientific context, several studies cite from non-peer-reviewed publications and offer translations of texts from religious or spiritual traditions. Researchers have quoted from traditional texts mentioning that meditation is “to still the fluctuations of the mind” (Bærentsen et al., 2010), but ignore situating the neuroscience findings in a philosophical premise and in the larger context of the mind-body problem. Meditation research findings have not been discussed in the larger philosophical contexts of how and where the fit is, vis-a-vis the current notions of brain-mind identity or supervenience theory that forms the bedrock of cognitive neuroscience today (Damasio, 1994; Feldman Barrett, 2009). Instead of ignoring the philosophy, it is essential to revisit the underlying ontological positions from the philosophical doctrines of meditation practice. It is important to note that there is no implication that the investigation of neural correlates implies any specific stand on the mind-body problem, nor that any particular stance would be useful.

## Summary and Conclusion

A large and growing body of work is being carried out that aims to provide a framework of functional and structural brain activity associated with meditation practice. A wide variety of techniques and methods are collectively termed as “meditation” with mixed and at times contradictory findings reported within the neuroscientific literature. It seems that more than a confusion of labels, there is the lack of a theoretical foundation – a philosophical grounding – for the neuroscience of meditation to develop firm roots in order to flourish and bear fruit. While most meditation research has explored the psychological and neuroscientific perspective, little work has been carried out at the philosophico-literary level.

As outlined in this article, the lack of understanding reflected in the definition issue impacts research design in this area, and contributes to other problems such as a scarcity of replication studies. This further exacerbates into a failure to address important questions like: *how* does meditation bring about changes at the neural, cognitive, and behavioral level? Prior to drawing any causal inference, Moonesinghe et al. (2007) have emphasized a vital need for replication studies and have re-iterated such an exercise to be the cornerstone of science. So far, there have been sparse attempts to consolidate research findings from a conceptual, psychological, and neural perspective. In a review, combining concepts like attention and emotional regulation, body and self-awareness, Holzel et al. (2011) explored a theoretical framework through which mindfulness meditation exerts its effects. This review, however, does not account for or include the philosophical underpinnings of meditation practice, neither in the neuroscientific context, nor in the traditional ontology.

In a study, Bærentsen et al. (2010) reported:

“the classical texts on meditation are in fine agreement with modern *systems theoretical approaches to understanding brain processes and consciousness*. Such theories regard consciousness and higher mental processes as a result of integrated activities in local areas of the brain, each contributing specific functions to the global activity pattern.” (p. 59, italics in original)

It should be noted, however, that the mind-body ontology in classical Yoga texts is dualist and is *not* in agreement with modern approaches that equate consciousness and mind as a result of brain activity. The foundational principles of Yoga are based on *Samkhya* philosophy which is explicitly dualist (i.e., consciousness and matter are ontologically separate entities; Sen Gupta, 1986). In fact, the whole effort of Yoga and meditation practice is to get rid of the “illusion” of mind-body identification achieved through moral, physical, and mental training (Dasgupta, 1924; Rao, 2011).

Various meditative practices are reported to induce a wide variety of altered states of consciousness. It is thus frequently claimed that the study of meditation will contribute to our general understanding of the neural basis of consciousness. However, how the phenomenology of meditation relates to the prevalent information-processing concept of the mind still remains an area open for examination and debate. Beyond developing a theory of physical embodiment, seeking causal explanations within a neural implementation might be at odds with the dualist framework of several foundational contemplative traditions. In the context of meditation research, before proceeding further with more empirical investigations, it is nonetheless essential to examine the philosophical context in which the meditation practices originate. In the absence of conceptual tools, theoretical models and the underlying ontological basis, the field may not progress beyond finding some psychological and neural correlates. With no clear external referents, or gold-standard measures with which to verify various meditative practices, there is a need for conceptual rigor in developing theoretical models consistent with the traditional ontology and to develop testable hypotheses in accordance with cognitive neuroscience methods. Ioannidis (2005, p. 698) has asserted that “greater the flexibility in designs, definitions, outcomes, and analytical modes in a scientific field, the less likely the research findings are to be true.”

## Possible Future Directions

Currently, a large variety of heterogeneous groups of practices focusing on attentional, mindfulness, relaxational, imagery-based interventions are currently subsumed under the same label “meditation.” As a possible step toward redressing this issue in the literature, paying due attention to the contextual and historical origins of meditation traditions is vital, if the definitions are to be understood (and translated) correctly. Instead of ignoring the first-person methodologies from traditional psychology, it might serve better, the cause of developing a comprehensive science of meditation, to integrate them with the third-person methodologies of neuroscience.

Sedlmeier et al. (2012) argue that the current state of theories on meditation does not allow us to derive very specific hypotheses, at least not for most of the dependent measures that have been studied in meditation research so far. In the absence of proper theoretical grounding, alternative explanations seem to overshadow the veracity and reliability of the results. Since, the dependent measures examined in the vast majority of studies still lack precision (see Sedlmeier et al., 2012), it is useful to explore the respective effects while being mindful of differences in the various techniques being studied. In the pursuit of measuring psycho-physical correlates of meditation, the psychological, physiological, and behavioral measures currently employed by researchers may not be specific to the particular meditation sub-type.

An in-depth study within the philosophico-literary aspects of meditation can help propose testable hypotheses within the scientific domain. Different stages of meditation have been extensively discussed in the traditional texts and post philosophico-literary deliberation, these could be useful to develop testable hypothesis within the neuroscientific domain. Comparative studies exploring differences between various approaches and practitioners from various traditions might serve to provide useful pilot data to delineate neurophysiological differences across the spectrum. Further, in agreement with suggestions by Sedlmeier et al. (2012), identification and isolation of the components of attention within various meditation methods, deriving predictions from different combinations of these components and investigating their impact by suitable measures could be a promising start.

Chiesa (2012) has re-iterated the overlap between the concept of mindfulness and related concepts of equanimity, ethics, wisdom, compassion etc. Further, exploration of context-effects, non-attentional components (such as sensory deprivation or enhancement methods, chaotic versus relaxed breathing) and moral disciplines could be very useful. Since, there are likely to be several stages involved in meditation sub-types, findings from single case studies as well as group comparisons might add valuable contributions to the field.

Neuroscience to-date has focused mainly on the third-person, neuro-behavioral side of the explanatory gap within the mind-body problem. By not giving sufficient attention to the ways in which meditation has been interpreted, developed, and expounded in the original sources, meditation researchers neglect and misunderstand important aspects of the method. A fruitful avenue of research might be to investigate aspects of meditation using a definition that could possibly do justice to both contemplative traditions and modern cognitive neuroscience. Here, the challenge for a comprehensive account of meditation may not be to show that the inferences from one or the other tradition are false, but rather how the perspectives adopted by these traditions might offer useful, complementary insights into the nature of the phenomena of mental training.

Attempts to include the phenomenological account of meditation will help ascertain and formulate a clear and operational definition for making progress. In agreement with Lutz and Thompson (2003), it might be useful to adopt a neurophenomenological approach that can be used to guide the study of physiological processes. An integration of traditional ontology, first-person phenomenological reports and neuroscientific findings will enable the development of more comprehensive models of the mind to help find common grounds for scientific research with the contemplative traditions. It will foster better study designs, leading to conclusive findings that could potentially be developed into systematic therapeutic interventions, besides fostering an interesting and important avenue of research into the mind-body problem.

## Conflict of Interest Statement

The author declares that the research was conducted in the absence of any commercial or financial relationships that could be construed as a potential conflict of interest.
